# Überwachung von COVID-19 durch Erweiterung der etablierten Surveillance für Atemwegsinfektionen

**DOI:** 10.1007/s00103-021-03303-2

**Published:** 2021-03-24

**Authors:** Luise Goerlitz, Kristin Tolksdorf, Udo Buchholz, Kerstin Prahm, Ute Preuß, Matthias an der Heiden, Thorsten Wolff, Ralf Dürrwald, Andreas Nitsche, Janine Michel, Walter Haas, Silke Buda

**Affiliations:** 1 Abteilung für Infektionsepidemiologie, Robert Koch-Institut, Berlin, Deutschland; 2 Abteilung für Infektionskrankheiten, Robert Koch-Institut, Berlin, Deutschland; 3 Zentrum für Biologische Gefahren und Spezielle Pathogene, Robert Koch-Institut, Berlin, Deutschland

**Keywords:** Syndromische Surveillance, Virologische Surveillance, Krankheitsschwere, SARS-CoV-2, Influenzapandemieplanung, Syndromic surveillance, Virological surveillance, Seriousness of disease, SARS-CoV-2, Influenza pandemic preparednesss

## Abstract

Im Rahmen der nationalen Influenzapandemieplanung wurden in Deutschland neben dem Meldewesen gemäß Infektionsschutzgesetz (IfSG) weitere Überwachungssysteme etabliert. Ziel dieser Systeme sind die Beschreibung, Analyse und Bewertung der Situation bei akuten respiratorischen Erkrankungen (ARE), die Identifikation der hauptsächlich zirkulierenden Atemwegserreger und die Beschreibung des zeitlichen Verlaufs. Seit Beginn der COVID-19-Pandemie wurden die Systeme erweitert, um auch Infektionen mit SARS-CoV‑2 erfassen zu können.

In diesem Beitrag werden drei verschiedene Surveillance-Systeme für ARE vorgestellt: GrippeWeb, die Arbeitsgemeinschaft Influenza mit dem SEED^ARE^-Modul (Sentinel zur elektronischen Erfassung von Diagnosecodes) und das Krankenhaus-Sentinel ICOSARI (ICD-10-code-basierte Krankenhaus-Surveillance schwerer akuter respiratorischer Infektionen). Mit diesen Systemen können ARE auf Bevölkerungsebene, im ambulanten und im stationären Bereich überwacht werden. Zusammen mit dem Monitoring der Mortalität liefern sie wichtige Hinweise zur Häufigkeit verschieden schwerer Krankheitsverläufe in der Bevölkerung. Um die Systeme für SARS-CoV‑2 zu erweitern, waren nur wenige Anpassungen notwendig.

Da die Falldefinitionen für ARE nicht geändert wurden, können in den beschriebenen Systemen historische Zeitreihen zum Vergleich herangezogen werden. Alle Systeme sind so aufgebaut, dass stabile und etablierte Bezugsgrößen für die Berechnung von wöchentlichen Anteilen und Raten zur Verfügung stehen. Dies ist eine wichtige Ergänzung zum Meldewesen gemäß IfSG, welches stark von Testkapazitäten und -strategien sowie veränderten Falldefinitionen abhängt. Die Surveillance-Systeme haben sich in der COVID-19-Pandemie auch im internationalen Vergleich als praktikabel und effizient erwiesen.

## Abstract (en)

As part of the national influenza pandemic preparedness, surveillance systems have been established in Germany in addition to the mandatory notifications according to the Protection Against Infection Act. The aim of these systems is the description, analysis, and evaluation of the epidemiology of acute respiratory infections (ARIs), the identification of the circulating viruses, and the trend. Since the beginning of the COVID-19 pandemic, the systems have been expanded to enable monitoring of infections with SARS-CoV‑2.

Three systems are presented: GrippeWeb, the primary care sentinel Arbeitsgemeinschaft Influenza with its electronic reporting module SEED^ARE^, and the ICD-10-based hospital sentinel ICOSARI. With these systems, ARIs can be monitored at the population, outpatient, and inpatient levels. In combination with the monitoring of mortality, these systems provide important information on the frequency of different stages of disease severity in the population. In order to expand the systems to SARS-CoV‑2, only a few adjustments were needed.

As the case definitions for ARIs were preserved, historical baselines of the systems can still be used for comparison. All systems are structured in such a way that stable and established reference values are available for calculating weekly proportions and rates.

This is an important addition to the mandatory reporting system of infectious diseases in Germany, which depends on the particular testing strategy, the number of tests performed, and on specific case definitions, which are adapted as required.

The surveillance systems have proven to be feasible and efficient in the COVID-19 pandemic, even when compared internationally.

Im Rahmen der Influenzapandemieplanung spielen Überwachungssysteme zur Beurteilung der aktuellen Situation eine zentrale Rolle. Nur mit einer realistischen Situationseinschätzung lassen sich Maßnahmen und Interventionen an die gegebenen Verhältnisse anpassen und dienen so in optimaler Weise dem Hauptziel jeder Pandemiebewältigungsstrategie: der Reduktion von Morbidität und Mortalität in der Bevölkerung [1].

Die meisten Pandemiepläne und Vorschläge für pandemieadaptierte Surveillance-Systeme sind konkret auf das Auftreten eines neuartigen Influenzavirus ausgerichtet, da es in der Vergangenheit nur Influenzapandemien mit erheblichen gesamtgesellschaftlichen Auswirkungen gegeben hat, so in den Jahren 1918, 1957, 1968 und 2009 [2, 3]. Das Auftreten der SARS-Epidemie (Severe Acute Respiratory Syndrome) im Jahr 2003 in China und Hongkong mit rascher weltweiter Verbreitung und die Ausbrüche von MERS (Middle East Respiratory Syndrome) seit 2012 auf der arabischen Halbinsel und in Südkorea verdeutlichten jedoch, dass nicht nur Influenzaviren ein großes pandemisches Potenzial besitzen, sondern virale, respiratorisch übertragene Erkrankungen zoonotischen Ursprungs generell [4, 5].

Surveillance-Systeme dienen der systematischen, kontinuierlichen Erhebung, Zusammenstellung, Analyse und Bewertung von Daten sowie der zeitnahen, kontinuierlichen Berichterstattung der Ergebnisse. Bei der Etablierung von Surveillance-Systemen sind die Repräsentativität der Datenquellen, das Erheben von Daten für die verschiedenen Schweregrade der Erkrankung und das Erfassen von Bezugsgrößen wichtige Qualitätskriterien. Für die Bewertung von Surveillance-Daten in einer Influenzapandemie oder einer durch andere Erreger hervorgerufenen Pandemie akuter respiratorischer Erkrankungen, gegen die in der Bevölkerung keine Immunität besteht, sind historische saisonale Daten zum Vergleich des Ausmaßes von Ausbreitung und Schwere unabdingbar.

Wissenschaftliche Studien sind dagegen zeitlich begrenzt und auf eine gezielte Fragestellung ausgerichtet. Ihre Ergebnisse werden im Allgemeinen in wissenschaftlichen Publikationen der Fachöffentlichkeit zur Verfügung gestellt. Die Ergebnisse aus Surveillance und Studien bilden gemeinsam einen maßgeblichen Teil der Informationen, die für eine kontinuierliche Risikoeinschätzung vor, während und nach einer Pandemie notwendig sind [1, 6–8].

Bei kontinuierlichen Surveillance-Systemen wird in Deutschland unterschieden zwischen dem Meldewesen gemäß Infektionsschutzgesetz (IfSG), in welchem meldepflichtige Erkrankungen und Erkrankungshäufungen detailliert erfasst werden, und der syndromischen Surveillance, die sich auf Symptomkombinationen stützt. Während die Daten aus dem Meldewesen immer auch im Kontext der bestehenden Teststrategie und der gültigen Falldefinitionen für bestimmte Erreger gewertet werden müssen, ermöglichen die syndromischen Surveillance-Systeme eine kontinuierliche und standardisierte Erfassung der Transmission und Krankheitslast durch ARE. Dabei ist eine Ergänzung der syndromischen Surveillance durch virologische Untersuchungen wichtig, um die aktuell zirkulierenden Erreger zu identifizieren, die in der beobachteten Situation das Geschehen bestimmen.

In diesem Beitrag werden drei verschiedene Surveillance-Systeme für akute Atemwegsinfektionen vorgestellt, die in Deutschland auch für den Fall einer Pandemie aufgebaut wurden: das Onlineportal GrippeWeb, die Arbeitsgemeinschaft Influenza mit dem SEED^ARE^-Modul (Sentinel zur elektronischen Erfassung von Diagnosecodes) und der begleitenden virologischen Surveillance und das Krankenhaus-Sentinel ICOSARI (ICD-10-code-basierte Krankenhaus-Surveillance schwerer akuter respiratorischer Infektionen). Die Anpassung dieser Systeme zur Überwachung der Erkrankungen mit dem pandemischen Coronavirus SARS-CoV‑2 wird beschrieben. Die Vorteile der Verwendung während der COVID-19-Pandemie werden dargestellt.

## GrippeWeb und GrippeWeb-Plus 2020

*GrippeWeb* ist das *Onlineportal, das in Deutschland die Aktivität akuter Atemwegserkrankungen beobachtet* und dabei Informationen aus der Bevölkerung selbst verwendet. Als Ergänzung zur Arbeit der Arbeitsgemeinschaft Influenza ging GrippeWeb im März 2011 online.

Die bei GrippeWeb registrierten Personen (Stand November 2020: rund 20.000) können dem Robert Koch-Institut (RKI) jede Woche anonym melden, ob sie (oder eines der im Haushalt lebenden minderjährigen Kinder) eine neu aufgetretene Atemwegserkrankung mit Symptomen wie Husten, Schnupfen, Halsschmerzen oder Fieber hatten oder ob dies nicht der Fall war. Die Daten von bis zu 9000 regelmäßig Meldenden gehen in die wöchentlichen Auswertungen ein. Wichtig sind die Meldungen auch dann, wenn keine Beschwerden auftraten. Denn nur so kann der Anteil der GrippeWeb-Teilnehmenden mit akuten Atemwegserkrankungen berechnet werden. Unterschieden wird zwischen akuten Atemwegserkrankungen im Allgemeinen (ARE) und Erkrankungen mit einer für Grippe typischeren Symptomatik (Influenza Like Illness, ILI).

Das System wurde erfolgreich validiert und konnte bereits nach wenigen Jahren seinen wichtigen Zusatznutzen zu den bis dahin etablierten Systemen zeigen [9]. Im Jahr 2016 wurde GrippeWeb im Rahmen einer Pilotierung durch eine virologische Komponente ergänzt, um auch auf Bevölkerungsebene einen Überblick über die zirkulierenden Atemwegserreger zu erhalten. Dabei wurden durch die Teilnehmenden selbst Proben abgenommen und am RKI auf typische Atemwegserreger untersucht (virologische Surveillance auf Bevölkerungsebene). Die Ergebnisse korrelierten sehr gut mit dem schon seit Jahrzehnten etablierten System der virologischen Surveillance der Arbeitsgemeinschaft Influenza [10]. Die Selbstabnahme respiratorischer Proben durch die Teilnehmenden konnte ohne größere Schwierigkeiten durchgeführt werden [10]. Die Erkenntnisse aus dieser Machbarkeitsstudie waren in vielerlei Hinsicht insbesondere zu Beginn der COVID-19-Pandemie nützlich, da Teile des Studienprotokolls und auch die Anleitung zur Probenselbstabnahme gut für andere COVID-19-Studien und Massenbeprobungen übernommen bzw. adaptiert werden konnten.

Basierend auf dieser Machbarkeitsstudie startete in GrippeWeb in der 13. Kalenderwoche (KW) 2020 die Surveillance auf respiratorische Atemwegserreger [11]. Dabei nimmt eine randomisierte Stichprobe von etwa 200 GrippeWeb-Teilnehmenden an einer mikrobiologischen Überwachung teil (GrippeWeb-Plus 2020; [10]). Seit der 13. KW 2020 entnehmen die Teilnehmenden bei sich selbst Proben aus der Nase und dem Gaumen und schicken diese an das RKI, wo sie auf 21 verschiedene Erreger, darunter auch SARS-CoV‑2, getestet werden. Über Zwischenergebnisse dieser Studie wurde in regelmäßigen Abständen in den GrippeWeb-Wochenberichten und im Situationsbericht des RKI zu COVID-19 berichtet.

Zusätzlich wurde die seit 2017 in Planung und Entwicklung befindliche GrippeWeb-App weiter vorangetrieben [12]. Zunächst wird das System vollständig neu programmiert für einen modernisierten Internetauftritt, der auch für mobile Endgeräte konzipiert sein wird. Mit der App könnten nach Überwindung der letzten technischen und datenschutzrechtlichen Hürden weit mehr GrippeWeb-Teilnehmende einfach und komfortabel ihre wöchentliche GrippeWeb-Meldung absetzen. Ein zusätzlicher Vorteil wäre die schnelle Information der Teilnehmenden zu pandemierelevanten Fragen (Pushbenachrichtigungen).

GrippeWeb lieferte von Beginn an wichtige Informationen zur Situation akuter Atemwegserkrankungen bei Kleinkindern, Jugendlichen und Erwachsenen. Diese wurden unter anderem im Rahmen der vom Deutschen Jugendinstitut in Kooperation mit dem RKI durchgeführten Corona-KiTa-Studie verwendet [13].

**Figure 1:**
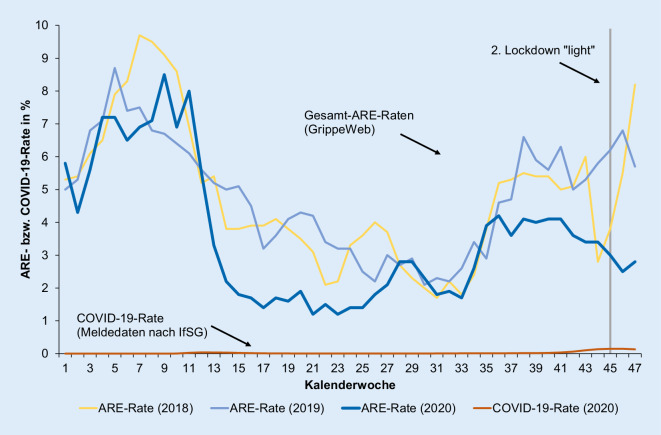

Insgesamt hat sich GrippeWeb bis zum Stand der Berichtserstellung im November 2020 als unverzichtbares Instrument der Überwachung akuter Atemwegsinfektionen erwiesen (Abb. 1). Alle anderen Surveillance-Systeme, die auf Informationen von Patienten im Gesundheitsversorgungssystem zurückgreifen, unterliegen naturgemäß den Änderungen und Wechseln im Konsultationsverhalten und in der Patientenlenkung, die sich in den unterschiedlichen Phasen der COVID-19-Pandemie durch Empfehlungen oder Verordnungen ergaben und weiterhin ergeben werden. Anhand der wöchentlichen Meldungen der GrippeWeb-Teilnehmenden können dagegen Aussagen zur Häufigkeit akuter Atemwegserkrankungen auf Bevölkerungsebene getroffen werden. Darüber hinaus bietet GrippeWeb auch zeitnähere Informationen, weil der Erkrankungsbeginn erfasst wird und nicht erst der Zeitpunkt des Aufsuchens des Gesundheitsversorgungssystems.

## Arbeitsgemeinschaft Influenza mit SEED^ARE^-Modul und virologischer Surveillance

Die *Arbeitsgemeinschaft Influenza (AGI)* blickt auf eine lange Geschichte zurück [14, 15]. Sie wurde 1992 gegründet und zunächst als öffentlich-private Partnerschaft durchgeführt. Für dieses deutschlandweit bekannte Sentinel-System für Influenza wurden Parameter entwickelt, die einen Überblick über die epidemiologische Situation der akuten Atemwegserkrankungen im Allgemeinen und der Influenza im Besonderen geben. Dieser Teil der Überwachung wird syndromische Surveillance genannt, da die beteiligten Arztpraxen eine Symptomkombination (= Syndrom) melden, die typisch für akute Atemwegserkrankungen ist. Hierbei kann das Syndrom entweder klinisch definiert werden (akute Pharyngitis, Bronchitis oder Pneumonie, jeweils mit oder ohne Fieber) oder über eine Kombination von ICD-10-Diagnosecodes wie im SEED^ARE^-Modul. Die syndromische Surveillance wird durch die virologische Untersuchung der Proben von Patienten mit akuten Atemwegserkrankungen begleitet (virologische Surveillance), welche das Nationale Referenzzentrum für Influenzaviren (NRZ) seit Beginn durchführt [16].

Im Jahr 2001 übernahm das RKI – im Kontext der Umsetzung des damals in Kraft tretenden neuen Infektionsschutzgesetzes – die wissenschaftliche Federführung der AGI.

Im Rahmen der Pandemieplanung wurde erkannt, dass die Überwachung der Influenza eine derart wichtige und zentrale Aufgabe in der Pandemiebewältigung darstellt, sodass sie vom Bund übernommen werden sollte. Im Gegensatz zur Überwachung der saisonalen Influenza wurde außerdem der Bedarf einer ganzjährigen Surveillance gesehen, da Influenzapandemien nicht unbedingt im Winterhalbjahr beginnen müssen. So wurde die seit 2006 zusätzlich durchgeführte Sommer-Surveillance von Anfang an aus öffentlichen Mitteln finanziert. Gerade im Frühjahr und Sommer 2009, als die pandemische Influenza A (H1N1) Deutschland erreichte, bildeten die Hintergrunddaten der Sommer-Surveillance eine wichtige Voraussetzung für eine fundierte Beurteilung der epidemiologischen Lage in Deutschland. Seit der Wintersaison 2009/2010 hat das RKI die AGI vollständig übernommen. Sie wird ausschließlich aus öffentlichen Mitteln finanziert. Die AGI ist seitdem eine Gemeinschaft der Sentinel-Praxen und des RKI. An der syndromischen Surveillance der AGI beteiligen sich mehr als ein Prozent der primärversorgenden Ärztinnen und Ärzte in Deutschland, womit eine Datengrundlage erreicht wurde, die auch international als repräsentativ erachtet wird, um auf nationaler Ebene statistische Auswertungen durchführen zu können.

Im Rahmen der technischen Weiterentwicklung der syndromischen Surveillance wurde ab 2006 ein System entwickelt, das elektronische Daten aus dem Arztinformationssystem (AIS) der Arztpraxen zur robusten und standardisierten Erfassung ambulanter ARE-Konsultationen nutzt. Bei dem *Sentinel zur elektronischen Erfassung von Diagnosecodes akuter respiratorischer Erkrankungen (SEED*^*ARE*^*)* werden über das AIS fallbasiert anonymisierte Daten von Patienten mit einer akuten Atemwegserkrankung anhand der ICD-10-Codes J00–J22, J44.0 und B34.9 (mit Angaben zu Alter, Geschlecht, Influenzaimpfung in der Praxis, Arbeitsunfähigkeit, Hospitalisierung) erfasst sowie die Anzahl aller Konsultationen in der Praxis, aggregiert nach sieben Altersgruppen, ermittelt [8]. Diese Daten werden einmal wöchentlich von den teilnehmenden Praxen verschlüsselt an das RKI übermittelt und im RKI analysiert. Das System liefert seit 2009 kontinuierlich und zuverlässig Daten. Nach erfolgreicher Validierung gehen diese erhobenen Daten seit der Saison 2012/2013 in die syndromische Surveillance und Berichterstattung der AGI ein [17].

Zu Beginn der COVID-19-Pandemie, als die Grippewelle der Saison 2019/2020 ihren Höhepunkt bereits überschritten hatte, lieferte das Überwachungssystem für den ambulanten Bereich wichtige Daten nicht nur zur Krankheitslast der Influenza. Es war auch ein wichtiges Instrument, das während des COVID-19-bedingten Lockdowns im Frühjahr 2020 die Wirksamkeit der nichtpharmakologischen Maßnahmen im ambulanten Bereich als Reduktion der akuten Atemwegserkrankungen und der Verkürzung der Influenzawelle um rund 2 Wochen exemplarisch zeigen konnte [18].

**Figure 2:**
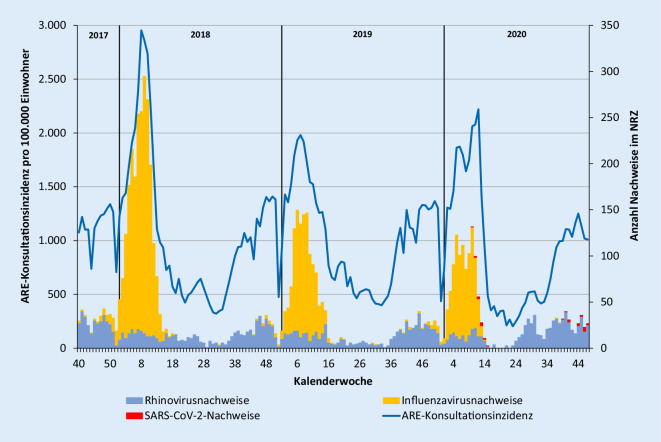

Dank der kontaktreduzierenden Maßnahmen und des verantwortungsbewussten Verhaltens großer Teile der Bevölkerung konnte eine unkontrollierte und ungebremste Verbreitung von SARS-CoV‑2 in der Bevölkerung, wie sie bei der saisonalen Zirkulation von Influenzaviren und dem fast ganzjährig zu beobachtenden Auftreten von Rhinoviren zu beobachten ist, bisher vermieden werden (Abb. 2).

Zusätzlich fließen Daten aus dem SEED^ARE^-System regelmäßig in die Berichterstattung der Corona-KiTa-Studie ein und stellen somit neben GrippeWeb eine weitere wichtige Datengrundlage zur Bewertung der Situation akuter Atemwegserkrankungen bei Kindern dar [13].

Zur Stärkung der syndromischen Surveillance wurden im Zeitraum von Juli und bis November 2020 in einer bis dahin einmaligen Aktion weitere SEED^ARE^-Praxen rekrutiert, sodass rund 280 neue Praxen aus dem primärversorgenden Bereich für die Teilnahme an diesem System gewonnen werden konnten. An der ARE- und Influenza-Surveillance der AGI haben sich bis November in der Saison 2020/2021 rund 735 registrierte Arztpraxen aktiv beteiligt, darunter rund 435 (60 %) über das SEED^ARE^-System. Ab dem 4. Quartal 2020 wurde das SEED^ARE^-System zudem um COVID-19-spezifische Codes erweitert (U07.1!, U07.2! und U99.0!). Die bestehende Erfassung der ICD-10-Codes J00–J22, J44.0 und B34.9 als ARE-Syndrom blieb dabei unverändert. Dies ermöglicht eine Kontinuität in der Datenerfassung, die das Fortbestehen historischer Zeitreihen sichert.

Die virologische Surveillance der AGI wird vom Nationalen Referenzzentrum für Influenzaviren durchgeführt. Es beteiligen sich Sentinel-Praxen aus der syndromischen Surveillance, die nach Kriterien für die geografische Lage der Praxis und nach Fachrichtung als repräsentativ angesehen werden. Die virologische Surveillance umfasste bis zum Januar 2020 die Untersuchung aller eingesandten Sentinel-Proben auf Influenzaviren, Rhinoviren, respiratorische Synzytialviren, humane Metapneumoviren und Parainfluenzaviren. Im Februar 2020 wurde die Untersuchung aller Sentinel-Proben auf SARS-CoV‑2 erweitert [19]. Seit dem Saisonbeginn 2020/2021 werden alle Sentinel-Proben zusätzlich noch auf saisonale humane Coronaviren untersucht.

## Krankenhaus-Surveillance ICOSARI und virologische SARI-Surveillance

Das RKI hat im Rahmen einer wissenschaftlichen Kooperation mit der HELIOS Kliniken GmbH ein kontinuierliches syndromisches Sentinel-Krankenhaus-Surveillance-System für schwere akute respiratorische Infektionskrankheiten (SARI) entwickelt. Das System basiert auf der Auswertung anonymer, fallbasierter Daten von ICD-10-Codes und wenigen zusätzlichen Prozeduren, wie z. B. Beatmung oder intensivmedizinische Behandlung (*ICOSARI-*Projekt: *ICD-10-code-basierte Krankenhaus-Surveillance schwerer akuter respiratorischer Infektionen*; [7, 8, 20]). Ziel des Projektes ist es, den zeitlichen Verlauf saisonaler Influenzawellen und anderer schwerer akuter Atemwegsinfektionen im akutstationären Bereich zeitnah abzubilden und die Krankheitslast durch Influenza, Pneumonie und SARI im stationären Bereich saisonal im Vergleich mit Vorsaisons und zu anderen Ländern einzuschätzen. Seit Beginn der COVID-19-Pandemie setzt sich auch das Europäische Zentrum für die Prävention und die Kontrolle von Krankheiten (ECDC) für den Aufbau einer europaweiten abgestimmten SARI-Surveillance ein, was gleichfalls den Empfehlungen der Weltgesundheitsorganisation (WHO) entspricht [2, 3, 21].

Ab der 40. KW 2015 (Influenzasaison 2015/2016) wurden wöchentlich Daten von Fällen mit respiratorischen Erkrankungsdiagnosen an das RKI gesendet. Seit der 3. KW 2017 werden die Ergebnisse aus der Krankenhaus-Surveillance zum Verlauf der Fallzahlen in 5 Altersgruppen im Influenza-Wochenbericht veröffentlicht. Die anonymisierten Datensätze aus dem Sentinel enthalten die ICD-10-codierten Entlassungsdiagnosen aller Patienten, die mit einer respiratorischen Erkrankung stationär in einer der teilnehmenden Kliniken hospitalisiert waren. Zur Einschätzung der Krankheitslast schwerer akuter respiratorischer Erkrankungen wurden die ICD-10-Codes aus den Gruppen J09 bis J22 (Influenza sowie akute respiratorische Erkrankungen der unteren Atemwege) ausgewählt. Das Sentinel umfasste seit 2014 maximal 83 Kliniken, von denen in Abhängigkeit von der Datenvollständigkeit zwischen 70 und 80 Kliniken in die wöchentliche Berichterstattung einbezogen wurden. Die Sentinel-Kliniken haben ihre Standorte in 13 von 16 Bundesländern und repräsentieren seit 2014 zwischen 5 % und 6 % der hospitalisierten Patienten in Deutschland.

Schon sehr früh im Verlauf der Pandemie (in KW 9/2020) konnten ICD-10-Diagnosen für COVID-19 im ICOSARI-System aufgenommen werden, ermöglicht durch die direkte Kooperation mit dem Datenzentrum des Krankenhausnetzwerks. Noch bevor schwere COVID-19-Fälle in Deutschland in größerem Maße registriert wurden, konnten zudem Informationen zu Pneumoniepatienten aus Deutschland, die während des Beginns saisonaler Grippewellen hospitalisiert wurden, mit Literaturangaben zu Patienten mit COVID-19 in China verglichen werden, sodass eine sehr frühzeitige Schwereeinschätzung von COVID-19-Erkrankungen möglich war [22].

Im Verlauf der ersten Monate wurde das Krankenhaus-Sentinel fortwährend für das zeitnahe Monitoring von hospitalisierten COVID-19-Patienten ausgeweitet. Die Häufigkeit der Datenabfragen wurde deutlich verdichtet, sodass mehr Aktualität und Verlässlichkeit der Daten erreicht werden konnten. Darüber hinaus ermöglichte die Auswertung von vorläufigen Daten noch nicht entlassener Patienten eine bessere Möglichkeit der zeitnahen Einschätzung des weiteren Geschehens. Durch eine bereits vorher gut etablierte Datenbasis war darüber hinaus auch ein direkter Vergleich des Krankheitsverlaufs von SARI-Patienten mit COVID-19-Diagnose und SARI-Patienten während früherer saisonaler Grippewellen möglich. Dabei konnte gezeigt werden, dass der Krankheitsverlauf bei Patienten mit COVID-19-Diagnose häufiger tödlich endete. Auch die Beatmungsdauer war bei SARI-Patienten mit COVID-19-Diagnose während der ersten COVID-19-Welle signifikant länger als bei SARI-Patienten in früheren Grippewellen [23].

**Figure 3:**
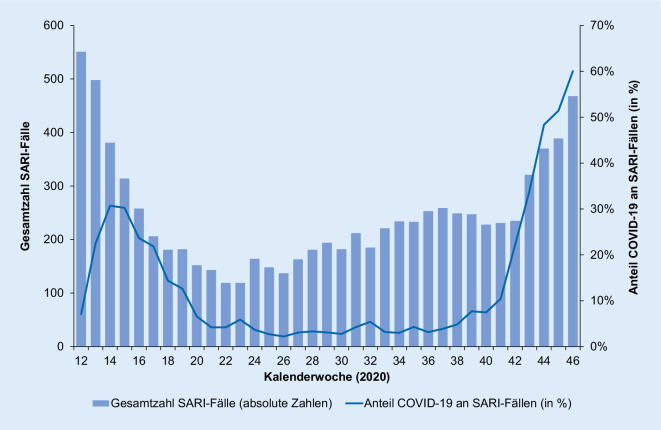

Im Gegensatz zur Aktivität akuter Atemwegserkrankungen in der Bevölkerung (GrippeWeb) und bei Arztbesuchen wegen akuter Atemwegserkrankungen (Arbeitsgemeinschaft Influenza) zeigt sich bei hospitalisierten Patienten mit schweren akuten respiratorischen Infektionen ein deutlicher Einfluss der COVID-19-Pandemie. Während in der ersten Phase der Pandemie der Anteil der SARI-Fälle mit COVID-19-Diagnose in den rund 70 Sentinel-Krankenhäusern bei maximal 31 % lag (14. und 15. KW 2020), stieg der Anteil der COVID-19-Fälle an den SARI-Fällen im Herbst auf rund 59 % (46. KW 2020; Abb. 3).

Seit der Saison 2019/2020 wird in Kooperation mit einem Krankenhaus in Berlin auch eine virologische SARI-Surveillance pilotiert. Vergleichbar mit dem Vorgehen im ambulanten Bereich wird diese ebenfalls vom Nationalen Referenzzentrum für Influenzaviren durchgeführt. Alle Krankenhaus-Sentinel-Proben werden auf das gleiche Erregerspektrum untersucht wie in der virologischen Surveillance der AGI, das heißt, dass auch die Untersuchung auf SARS-CoV‑2 zeitnah etabliert wurde. Im NRZ werden zudem weiterführende molekularbiologische Analysen an die Erregernachweise angeschlossen [19].

## Mortalitäts-Surveillance

Zur Beurteilung, inwieweit es in Deutschland zu ungewöhnlich hoher Sterblichkeit infolge der COVID-19-Pandemie kam oder noch kommt, wurden Systeme, die auch für die Beurteilung der Übersterblichkeit in Influenzawellen genutzt werden, eingesetzt [24, 25]. Diese werden im Rahmen des europäischen Netzwerks zum Mortalitätsmonitoring (EuroMOMO) koordiniert [26]. Im Gegensatz zu anderen europäischen Ländern, in denen insbesondere im Frühjahr 2020 Werte zur Übersterblichkeit beobachtet wurden, die weit über das bis dahin bekannte Ausmaß selbst starker saisonaler Grippewellen hinausgingen, konnte in Deutschland bisher keine derart hohe Übersterblichkeit konstatiert werden. Innerhalb von EuroMOMO können allerdings bisher nur die Daten und Auswertungen von zwei Bundesländern, Berlin und Hessen, genutzt werden, in anderen Bundesländern ist bisher noch kein zeitnahes Monitoring der Mortalität etabliert. Daneben wurde vom Statistischen Bundesamt (Destatis) in Kooperation mit dem RKI eine Sonderauswertung zu den wöchentlichen Sterbezahlen in Deutschland erstellt [27], die den aktuellen Verlauf der Gesamtmortalität im Vergleich zu den gemeldeten COVID-19-Todesfällen zeigt. Hier zeigte sich, dass die Zahl der dokumentierten Sterbefälle bei COVID-19-Fällen ähnlich hoch ist wie die beobachtete Übersterblichkeit in Deutschland. Dies deutet auf eine relativ gute Erfassung dieser Sterbefälle im Meldesystem nach IfSG hin. Ab November 2021 wird es eine bundesweite und flächendeckende, zeitnahe Mortalitäts-Surveillance am RKI geben [28].

## Fazit und Ausblick

Es konnten Systeme, die für die Überwachung saisonaler Grippewellen genutzt werden und im Rahmen der Influenzapandemieplanung in Deutschland, Europa und weltweit etabliert wurden, relativ leicht auch zur Überwachung von COVID-19 adaptiert werden. Viele Grundprinzipien der Überwachung zur Transmission, zum Krankheitsverlauf und dem altersabhängigen Risiko für schwere Krankheitsverläufe konnten übernommen oder ohne zu großen Aufwand adaptiert werden. Andere Staaten mit engen, erregerspezifischen Falldefinitionen und einer sehr fokussierten Ausrichtung auf Influenzaerkrankungen konnten die Influenzaüberwachung einerseits mangels entsprechender Ressourcen nicht fortführen und mussten andererseits ganz neue Überwachungssysteme etablieren, sodass historische Vergleichsdaten fehlen.

Im Gegensatz dazu waren die Influenzaüberwachungssysteme in Deutschland schon seit Beginn der Surveillance-Aktivitäten durch das breite Spektrum der abgedeckten akuten Atemwegsinfektionen sehr flexibel gestaltet. Auch in einer Influenzapandemie ist zu Beginn nicht bekannt, welche Altersgruppen besonders betroffen sind, welche Atemwegssymptome im Vordergrund stehen und welche Faktoren zu schweren Krankheitsverläufen beitragen. Für eine Erfassung im Meldewesen müssen zunächst der Verdacht, die Erkrankung und der Tod an einer Erkrankung und die Meldung eines Labornachweises eines neuartigen Erregers gesetzlich meldepflichtig werden. Anschließend werden Fälle durch Testung und Meldung in das Meldesystem gemäß Infektionsschutzgesetz überführt. Der anfangs notwendige Aufbau von Testkapazitäten hat zu Beginn eine Untererfassung der Fallzahlen über das Meldesystem bedeutet. Dagegen ermöglichten die bereits etablierten syndromischen Surveillance-Systeme auch vor der spezifischen Erweiterung um COVID-19-Diagnosecodes eine Einschätzung der Krankheitslast akuter Atemwegserkrankungen auf den verschiedenen Ebenen der Gesundheitsversorgung sowie auf Bevölkerungsebene. Selbst als aus Deutschland noch keine Daten zur Krankheitsschwere von COVID-19 vorlagen, konnten historische Daten mit Literaturangaben aus dem ersten betroffenen Land, der Volksrepublik China, für eine erste Risiko- und Schwereeinschätzung genutzt werden [22]. Die stabile Fortführung der Datenreihen aus der syndromischen Surveillance hat sich im Verlauf der SARS-CoV-2-Pandemie auch als wirksames Mittel erwiesen, um die Auswirkung von Anpassungen in der Teststrategie und im Meldesystem abschätzen zu können. Grundsätzlich sollten syndromische Surveillance-Systeme immer durch virologische Surveillance-Aktivitäten mit einem angepassten Erregerpanel begleitet werden, wie es bei der Arbeitsgemeinschaft Influenza der Fall ist. Zusätzlich zu den Meldungen gemäß Infektionsschutzgesetz, die nach wie vor die zentrale Daten- und Informationsquelle darstellen, wurden weitere erregerspezifische Systeme neu aktiviert, die sich auch zur Überwachung von saisonaler und/oder pandemischer Influenza bewährt haben [29, 30].

Der große Vorteil der Anpassung und Fortentwicklung bereits bestehender Surveillance-Systeme bei gleichzeitiger Beibehaltung historischer Datenreihen liegt in den Vergleichsmöglichkeiten, die die Zahl der Erkrankungen und die Krankheitsschwere in Relation zu bereits bekannten akuten respiratorischen Erkrankungen setzt.
